# Impact of the change of copay policy in Medicare Part D on zoster vaccine uptake among Medicare beneficiaries in a managed care organization

**DOI:** 10.1186/s12913-017-2441-7

**Published:** 2017-07-21

**Authors:** Rulin C. Hechter, Lei Qian, Songkai Yan, Yi Luo, Girishanthy Krishnarajah, Hung-Fu Tseng

**Affiliations:** 1 Department of Research & Evaluation, Kaiser Permanente Southern California, 100 South Los Robles Avenue, 2nd Floor, Pasadena, CA 91101 USA; 2 US Health Outcomes & Epidemiology - Vaccines, GSK, 5 Crescent Drive, Philadelphia, PA 19112 USA; 3 CSL Behring, 1020 First Avenue, King of Prussia, PA 19406 USA

**Keywords:** Zoster, Vaccination, Medicare, Part D, Copay

## Abstract

**Background:**

Kaiser Permanente Southern California (KPSC) adopted the Medicare Part D Tier-6 with zero patient copay for zoster vaccination in 2012. We assessed the impact of the implementation on zoster vaccination rate (GSK study identifier: HO-13-14,182).

**Methods:**

Zoster vaccination rate was examined among an open cohort of ≥65-year-old Medicare Part D beneficiaries during 01/01/2008–06/30/2014, compared to ≥65-year-old commercial health plan members and 60–64-year-old members. The demographics, vaccination records, and insurance and benefit type were confirmed through KPSC electronic medical record databases. Person-time based vaccination rate was calculated for each observation interval (calendar month or year). The changes in annual rates in one year pre- (2011) and post- (2012) Tier-6 implementation were compared in a difference-in-difference analysis. Linear spline Poisson regression models were fitted to compare the secular trend of monthly rates during pre and post Tier-6 implementation (01/2012).

**Results:**

Zoster vaccination rate increased in Medicare Part D beneficiaries after the implementation of zero copay. The increase in annual vaccination rate from 2011 to 2012 was marginally higher in Medicare Part D beneficiaries but not statistically significant (difference in rate ratio [RR] = 0.04, *p* > 0.05) compared to commercial health plan members. Among non-Hispanic white members, the difference of RR was 0.09 (*p* = 0.020) between Medicare Part D beneficiaries and ≥65-year-old commercial plan members, and it was 0.08 (*p* = 0.034) compared to 60–64-year-old commercial plan members. In secular trend analysis, we did not observe significant increase in overall and race stratified zoster vaccination rate attributable to the implementation of the Tier-6.

**Conclusions:**

The impact of Tier-6 on zoster vaccination was not substantial in elderly Medicare Part D beneficiaries in this population where a lower than average copay ($20 to $40) was applied prior to the Tier-6 implementation. Further research is necessary to explore the numerical relationship between vaccination and amount of copay.

**Trial registration:**

**GSK study identifier:** HO-13-14,182.

**Electronic supplementary material:**

The online version of this article (doi:10.1186/s12913-017-2441-7) contains supplementary material, which is available to authorized users.

## Background

Despite the United States (US) Advisory Committee on Immunization Practices (ACIP) recommendation of a single dose of zoster vaccine for routine use among all persons aged ≥60 years [1], zoster vaccine coverage rate has remained low. According to the National Health Interview Survey (NHIS) data, the proportion of people aged ≥60 years who ever received zoster vaccine was 6.7% (95% confidence interval [CI]: 5.9–7.5) in 2008 and 20.1% (95% CI: 19.1–21.2) in 2012 [2–5]. Zoster vaccine is covered by Medicare Part D benefit [6] and Medicare beneficiaries were required to pay a cost sharing for the injectable vaccines covered by Part D during 2008–2011. The cost sharing for the zoster vaccine and the complexity of reimbursement for the vaccine through Medicare Part D have been identified as important barriers to vaccine uptake among the elderly [7]. In 2011, as an effort to remove the cost sharing barrier to vaccine uptake, the Centers for Medicare & Medicaid Services (CMS) introduced a new tier (Tier-6) in Part D to allow the costs for all injectable Part D vaccines (including the zoster vaccine) and associated administration fees to be covered by the Tier-6 with zero cost sharing by the patients. To date, the impact of the implementation of Tier-6 on zoster vaccination uptake in persons with Part D coverage (those aged ≥65 years) has not been evaluated. We sought to assess the impact of the introduction of Tier-6 in Medicare Part D on zoster vaccination rate among persons aged ≥65 years with Medicare Part D coverage.

## Methods

### Study setting

We conducted an analysis in adults enrolled in Kaiser Permanente Southern California (KPSC). KPSC is a large managed care organization that provides integrated care to over 4 million health plan members in Southern California, about 10% of KPSC members are covered by Medicare plans and the majority of these Medicare members have the Part D drug benefit. KPSC is one of the first health plans in the US that adopted the Tier-6 coverage benefit for the Medicare members. Starting from January 1st, 2012, all KPSC Medicare members with the Part D drug benefit could receive the zoster vaccine with zero copay for vaccination at a nurse or physician visit, whereas the copay amount was between $20–40 before the implementation of the Tier-6. KPSC has a comprehensive electronic medical record (EMR) system that captures demographics, immunization history, health care utilization, and members’ health plan type. Rigorous data quality and validation work on immunization records have routinely been conducted.

### Study design and study population

This observational study (GSK study identifier: HO-13-14,182) assessed the impact of Tier-6 on zoster vaccination uptake among Medicare Part D beneficiaries aged ≥65 years. Three cohorts of adults enrolled in KPSC health plans any time from January 1st, 2008 through June 30th, 2014 were identified to evaluate the difference in the change of vaccination rate during pre- and post- Tier-6 periods: one target cohort (i.e., ≥65-year-old Medicare Part D beneficiaries) and two comparison cohorts (comparison cohort 1: ≥65-year-old KPSC commercial plan members and comparison cohort 2: 60–64-year-old KPSC commercial plan members). The two comparison cohorts were chosen to account for the effects of changes in zoster vaccine supply, direct-to-consumers (DTC) advertising, and public awareness of zoster vaccine during the study period. The cost sharing for the zoster vaccine was applied to the Medicare Part D beneficiaries in the target cohort before January 1st, 2012, while there was no copay for zoster vaccine charged to the commercial plan members in the two comparison cohorts during the entire study period.

Zoster vaccine records were ascertained through the KPSC EMR database. Zoster vaccination rates in the target cohort and the two comparison cohorts were calculated for the one year pre- (2011) and post- (2012) Tier-6 implementation periods and each observation interval (i.e., calendar month) during the study period. The eligibility for vaccination was evaluated for each individual at the entry into the open cohort during each observation interval. Individuals who had received the zoster vaccine any time prior to January 1st, 2008 were excluded. An individual can continue to contribute person-time in multiple observation intervals prior to the receipt of the zoster vaccination. An individual would stop contributing person-time and be excluded from the following observation intervals if they either received the vaccine during the current observation interval, moved out of the age range for the cohort (for 60–64-year-old commercial plan members cohort only), or exited the cohort due to loss to follow-up.

### Statistical analysis

#### Descriptive analysis

Socio-demographic characteristics (e.g., age, sex, neighborhood socioeconomic status (SES)) and vaccination status during the study period were summarized using descriptive statistics, by pre- and post- Medicare Part D Tier-6 implementation periods, for the target cohort and two comparison cohorts. Frequency and proportion were calculated for categorical variables such as gender and race/ethnicity. Mean (standard error) and median (ranges) were reported for continuous variables. Distribution of age, gender, race/ethnicity, and neighborhood SES estimates were presented for the target cohort and each comparison cohort. Monthly and annual vaccination rates were defined as (Number of eligible subjects who received zoster vaccine during the observation interval) / (Total person-time contributed by the subjects who were eligible for the vaccine during the observation interval). A subject was considered eligible for zoster vaccine during an observation interval if he/she did not receive the vaccine prior to the entry into the cohort at that specific observation interval. Tables of the annual zoster vaccination rates and graphs of the monthly vaccination rates during the study period were generated to describe the trends of the vaccination rate changes in the three cohorts in the pre- and post- Tier-6 periods.

#### Difference-in-difference analysis

We conducted difference-in-difference (DID) analyses on the annual vaccination rates in the target cohort and the two comparison cohorts during the year pre- (2011) and the year post- (2012) Tier-6 implementation. The DID method calculates the effect of the intervention (i.e., the implementation of Tier-6 in this study) on an outcome (i.e., vaccination in this study) by comparing the average change in the outcome variable pre- and post- intervention in the target group to the average change pre and post treatment in a comparison group. This method is able to adjust for non-time varying confounders within each group and time varying confounders that occur in both target and comparison groups. The DID analysis measures the change in the differences between the target and comparison groups over time. We calculated the difference in the change in zoster vaccination rate between the eligible ≥65-year-old adult members with Medicare Part D coverage at KPSC and the two commercial health plan cohorts (i.e., ≥65-year-old KPSC commercial plan members and those of 60–64 years, respectively) during the same 12-month pre- and post- implementation of the Tier-6. The ratio and the difference of the rate ratios and associated 95% CI were calculated and compared between the target cohort and the two comparison cohorts.

## Secular trend analysis

To account for the potential impact of time varying confounders during the study period (such as vaccine supply shortage or promotion of the vaccine in primary care) and to control for baseline trend of vaccine uptake, a secular trend analysis was conducted using monthly vaccination rate data, where age and the eligibility for vaccine of interest were updated at the date of entry into the open cohort during each calendar month (i.e., observation interval). The vaccination status for each eligible individual was updated for each calendar month and the vaccination rates were calculated among the total person-time contributed by eligible individuals during each calendar month. We used linear spline Poisson regression model to fit separate linear spline curves for each cohort to compare the secular trends of monthly vaccination rates in the target cohort to the comparison cohorts. A pre-specified change point at month 01/2012 was defined in the model to indicate the time point of the Tier-6 implementation in KPSC. The difference in slope changes immediately pre- and post- Tier-6 implementation (slope of year 2012 vs. 2011) between Medicare Part D cohort and the two comparison cohorts were tested among the overall study sample as well as by race/ethnicity subgroup.

All analyses were conducted by using *SAS* (version 9.3 for Windows, SAS Institute, Cary, NC).

## Results

We identified more than 160,000 individuals in each study cohort in each year during the study period. Demographic characteristics were generally comparable across the study period among the three study cohorts (except that subjects in the comparison cohort 2 were younger by definition). Table 1 presents the patient characteristics for 2011 and 2012. The largest ethnic group was non-Hispanic white, comprising about 50% in the Medicare Part D and the ≥65-year-old commercial plan cohorts, and over 40% in the 60–64-year-old commercial plan cohort. However, this percentage decreased slightly and steadily each year during 2008–2014, while the percentage of Hispanic members increased steadily each year in all study cohorts (data not shown). In general, members of the three cohorts in this study resided in a neighborhood with relatively high levels of education attainment and household income.

**Table 1:** Demographic characteristics of the study cohorts

haracteristics	Medicare Part D (65+)		Commercial (65+)		Commercial (60–64)	
	2011 (*n* = 175,524)	2012 (*n* = 192,080)	2011 (*n* = 179,334)	2012 (*n* = 181,927)	2011 (*n* = 197,653)	2012 (*n* = 200,517)
Age in years, mean	73.91	73.64	72.56	72.38	61.47	61.48
Median (Q1;Q3)	72 (68;79)	72 (67;78)	70 (66;77)	70 (66;77)	61 (60;63)	61 (60;63)
Sex, n (%)						
Female	98,629 (56.2)	107,000 (55.7)	91,193 (50.9)	92,312 (50.7)	101,613 (51.4)	103,114 (51.4)
Male	76,895 (43.8)	85,080 (44.3)	88,141 (49.1)	89,615 (49.3)	96,040 (48.6)	97,403 (48.6)
Race/Ethnicity, n (%)^a^						
Non-Hispanic white	95,558 (54.4)	101,282 (52.7)	90,141 (50.3)	89,986 (49.5)	88,971 (45.0)	89,435 (44.6)
Non-Hispanic black	11,837 (6.7)	12,830 (6.7)	28,247 (15.8)	28,513 (15.7)	20,781 (10.5)	21,243 (10.6)
Hispanic	46,324 (26.4)	53,092 (27.6)	33,975 (19.0)	35,678 (19.6)	48,384 (24.5)	50,986 (25.4)
Asian/Pacific Islanders	14,814 (8.4)	16,631 (8.7)	17,256 (9.6)	17,940 (9.9)	21,825 (11.0)	22,226 (11.1)
Others/unknown	6991 (4.0)	8245 (4.3)	9715 (5.4)	9810 (5.4)	17,692 (9.0)	16,627 (8.3)
Neighborhood education, mean (percentage with college and higher), %	58.8	58.09	59.86	58.82	59.44	58.44
Median (Q1;Q3)	60.86 (44.06;75.21)	59.63 (43.54;73.49)	61.80 (46.42;75.16)	60.31 (45.76;73.20)	61.53 (45.38;75.35)	59.83 (44.88;73.26)
Mean Neighborhood household income in $	64,482	64,057	65,540	65,275	66,720	66,274
Median (Q1, Q3)	59,583 (43,710;80,371)	59,447 (43,542;79,419)	61,101 (45,170;81,636)	61,143 (45,282;80,682)	62,266 (45,926;82,639)	62,114 (45,805;81,866)

^a^Sum of percentages may not be 100% due to rounding

n, number of subjects in each category; Q1, first quartile; Q3, third quartile

**Fig. 1:**
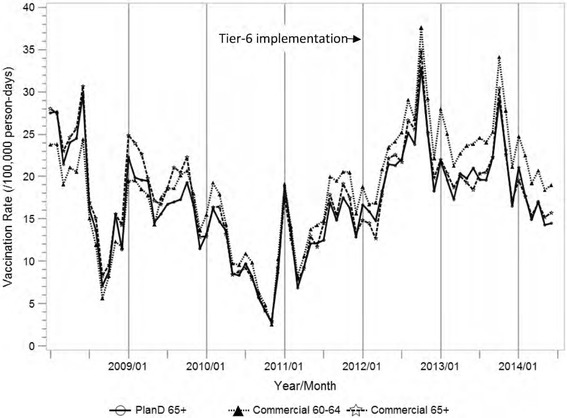
Monthly zoster vaccination rate from 2008 to 2014

There were no significant differences in the trend of monthly zoster vaccination rates among the three cohorts prior to the Tier-6 implementation despite differences in copay policy (Fig. 1). Zoster vaccination rates increased in all three study cohorts after January 2012, and the increase was slightly greater in males than in females in all study cohorts. Table 2 shows the changes in zoster vaccination rates for the three cohorts, from 2011 to 2012.

**Table 2:** Change in zoster vaccination rate (per 100,000 person-days) from 2011 to 2012

	Zoster vaccine uptake rate (1/100,000)								
Characteristics	Medicare Part D (65+)			Commercial (65+)			Commercial (60–64)		
	2011 (*n* = 175,524)	2012 (*n* = 192,080)	Difference (2012/2011)	2011 (*n* = 179,334)	2012 (*n* = 181,927)	Difference (2012/2011)	2011 (*n* = 197,653)	2012 (*n* = 200,517)	Difference (2012/2011)
Overall	13.64	21.35	1.57	14.14	21.54	1.52	15.93	24.23	1.52
Age									
60–64	N/A	N/A	N/A	N/A	N/A	N/A	15.93	24.23	1.52
65–69	17.97	27.81	1.55	17.04	25.26	1.48	N/A	N/A	N/A
70–74	14.21	21.75	1.53	14.53	22.43	1.54	N/A	N/A	N/A
75+	9.93	15.63	1.57	10.48	16.27	1.55	N/A	N/A	N/A
Sex									
Female	14.46	21.94	1.52	15.25	22.63	1.48	18.63	27.47	1.47
Male	12.59	20.61	1.64	12.98	20.40	1.57	13.08	20.82	1.59
Race/Ethnicity									
Non-Hispanic white	15.67	25.48	1.63	17.27	26.46	1.53	21.27	32.79	1.54
Non-Hispanic black	8.48	13.75	1.62	9.49	14.50	1.53	9.44	15.20	1.61
Hispanic	9.63	14.30	1.48	10.75	16.24	1.51	9.85	16.13	1.64
Asian/Pacific Islanders	19.17	26.27	1.37	15.51	23.96	1.54	18.94	25.77	1.36
Others/unknown	9.33	18.26	1.96	7.60	11.76	1.55	9.32	13.14	1.41

n, number of subjects in each category

The DID analysis results are showed in the Table 3 (Medicare Part D cohort vs. ≥65-year-old commercial plan members) and Table 4 (Medicare Part D cohort vs. 60–64-year-old commercial plan members). We observed an increase of 0.04 in rate ratio from 2011 to 2012 comparing Medicare Part D cohort vs. ≥65-year-old and 60–64-year-old commercial plan cohorts (*p* = 0.178 and 0.145 respectively, not statistically significant). In non-Hispanic white members, difference of rate ratio was 0.09 (*p* = 0.020) and 0.08 (*p* = 0.034) comparing Medicare Part D cohort vs. ≥65-year-old and 60–64-year-old commercial plan cohorts, respectively. In Hispanic members, there was no statistical significant difference of rate ratio between Medicare Part D cohort and the ≥65-year-old commercial plan cohort; however a statistically significant rate ratio difference of −0.15 (*p* = 0.038) was observed between Medicare Part D cohort vs. the 60–64-year-old commercial plan cohort.

**Table 3:** Comparison of zoster vaccination rate between Medicare Part D members and commercial plan members aged ≥65^a^

Characteristics	Medicare Part D (65+)	Commercial (65+)	Difference-in-difference		
	Rate ratio (2012/2011)	Rate ratio (2012/2011)	Ratio of rate ratio	*p*-value^b^	Difference of rate ratio
Overall	1.57	1.52	1.03 (0.99;1.07)	0.178	0.04 (−0.02;0.10)
Age					
60–64	N/A	N/A			
65–69	1.55	1.48	1.04 (0.99;1.11)	0.141	0.07 (−0.02;0.15)
70–74	1.53	1.54	0.99 (0.92;1.08)	0.853	−0.01 (−0.14;0.12)
75+	1.57	1.55	1.01 (0.94;1.09)	0.732	0.02 (−0.10;0.14)
Sex					
Female	1.52	1.48	1.02 (0.97;1.08)	0.411	0.03 (−0.05;0.11)
Male	1.64	1.57	1.04 (0.98;1.11)	0.187	0.07 (−0.03;0.16)
Race/Ethnicity					
Non-Hispanic white	1.63	1.53	1.06 (1.01;1.12)	0.020	0.09 (0.01;0.18)
Non-Hispanic black	1.62	1.53	1.06 (0.90;1.24)	0.467	0.09 (−0.18;0.36)
Hispanic	1.48	1.51	0.98 (0.89;1.08)	0.721	−0.03 (−0.18;0.13)
Asian/Pacific Islanders	1.37	1.54	0.89 (0.79;1.00)	0.050	−0.17 (−0.36;0.01)
Others/unknown	1.96	1.55	1.26 (1.00;1.61)	0.054	0.41 (−0.05;0.87)

^a^ Per 100,000 person-days and from 2011 to 2012

^b^ Calculated using linear spline Poisson regression model

**Table 4:** Comparison of zoster vaccination rate between Medicare Part D members and commercial plan members aged 60-64^a^

Characteristics	Medicare Part D (65+)	Commercial (60–64)	Difference-in-difference		
	Rate ratio (2012/2011)	Rate ratio (2012/2011)	Ratio of rate ratio	*p*-value^b^	Difference of rate ratio
Overall	1.57	1.52	1.03 (0.99;1.07)	0.145	0.04 (−0.02;0.10)
Age					
60–64	N/A	1.52			
65–69	1.55	N/A			
70–74	1.53	N/A			
75+	1.57	N/A			
Sex					
Female	1.52	1.47	1.03 (0.98;1.08)	0.265	0.04 (−0.03;0.12)
Male	1.64	1.59	1.03 (0.97;1.09)	0.358	0.05 (−0.05;0.15)
Race/Ethnicity					
Non-Hispanic white	1.63	1.54	1.05 (1.00;1.11)	0.034	0.08 (0.00;0.16)
Non-Hispanic black	1.62	1.61	1.01 (0.85;1.19)	0.939	0.01 (−0.28;0.30)
Hispanic	1.48	1.64	0.91 (0.83;0.99)	0.038	−0.15 (−0.30;0.00)
Asian/Pacific Islanders	1.37	1.36	1.01 (0.90;1.13)	0.905	0.01 (−0.15;0.17)
Others/unknown	1.96	1.41	1.39 (1.13;1.71)	0.002	0.55 (0.15;0.95)

^a^ Per 100,000 person-days and from 2011 to 2012

^b^ Calculated using linear spline Poisson regression model

The secular trend analysis did not show significant increase in overall and race-stratified zoster vaccination rates attributable to Tier-6 implementation in Medicare Part D cohort. The difference in trend slope changes (from 2011 to 2012) were not statistically significant between the Medicare Part D cohort and the two comparison cohorts: it was −0.004 (95% CI: -0.013-0.004, *p* = 0.305) between the Medicare Part D cohort and the 60–64-year-old commercial cohort, and it was −0.001 (95% CI: -0.010-0.007, *p* = 0.783) between the Medicare Part D cohort and the ≥65-year-old commercial cohort.

## Discussion

The findings from this study show that the distribution of age, gender, and neighborhood SES among patient population at KPSC is generally stable for both Medicare beneficiaries and commercial health plans members during the study period. The pattern of the changes in zoster vaccine uptake rate is similar across the three groups and seems to correspond with the timing of the vaccine shortage and supply restoration (the national zoster vaccine shortage began in July 2008 and was resolved by December 2011). In general, the uptake rate among 60–64-year-old commercial health plan members was slightly higher than that among ≥65-year-old Medicare Part D beneficiaries. After the implementation of the Tier-6, the vaccination rate increased by approximately 57% in 2012 in comparison to 2011 among ≥65-year-old Medicare Part D beneficiaries. However, a similar magnitude of increase in the vaccination rate was also observed among the commercial plan members (52% in both ≥65-year-old and 60–64-year-old commercial health plan comparison cohorts). The observed increase in the commercial plan members may partially be owed to the zoster vaccine supply restoration around January 2012.

Although the difference in rate ratio between the ≥65-year-old Medicare Part D cohort and the two comparison cohorts was not statistically significant among the overall sample in the DID analysis, a moderate and statistically significant increase in rate ratio was observed in non-Hispanic white, suggesting the Medicare Part D Tier-6 might have a greater impact on the zoster vaccine uptake among non-Hispanic white Medicare beneficiaries than the other racial/ethnic subgroups. However, the finding wasn’t confirmed by the secular trend analysis after we further controlled for the baseline trend of the vaccination rate.

Nevertheless, the findings from both DID analysis on annual vaccination rates and the secular trend analysis on the trend of vaccine uptake using monthly data both suggested that the overall impact of Tier-6 on zoster vaccine uptake among elderly Medicare Part D beneficiaries is not substantial in health plan members of an integrated health care organization. One study found that around 50% of zoster vaccinations from various Medicare Part D plans required a patient copay amount of $76 to $100 [8]. However, the lower copay for the zoster vaccine at KPSC before the implementation of the Tier-6 might have had a very small influence on the decision-making around receiving the zoster vaccination among the Medicare Part D members at KPSC prior to the Tier-6 implementation. This is substantiated by the fact that before the introduction of Tier-6, the uptake rates were similar across the three cohorts irrespective of the copay amount. Therefore the relatively lower vaccination rate we observed in this study population before the implementation of Tier-6 was less likely to be affected by a $20–40 copay, but rather by the national zoster vaccine supply shortage before 2012. There is evidence that showed the amount/level of the cost-sharing or copay is associated with the health care services uptake and utilization [7, 9–13]. Thus, the introduction of the Tier-6 in Medicare Part D coverage may have a larger impact on the zoster vaccine uptake in other health care settings where the cost-sharing was substantially higher prior to the Tier-6 implementation.

This study has several strengths. To our knowledge, this is the first large population-based study to assess the potential impact of the introduction of Tier-6 in the Medicare Part D benefit on the zoster vaccine uptake among elderly Medicare beneficiaries. The vaccination records were ascertained through a large EMR system at KPSC, which mitigated recall bias that is often inherited in many survey studies that largely rely on patient self-reported vaccination data. By using the EMR data, we were able to include all eligible individuals in the analysis, which minimized selection bias that could be potentially caused by low response rates or by using a convenience sample in surveys. In this study, we used two methods, DID and secular trend analysis and got similar results. By using the secular trend analysis, we were able to not only adjust for the baseline vaccination rate across the target and two comparison cohorts, but also adjust for the trend of uptake at the baseline across the three study cohorts.

### Limitations

This study has a few limitations. First, the zoster vaccination history was ascertained based on the vaccine records in EMR. The records for those individuals who received zoster vaccine prior to their enrollment into the KPSC health plans or who received the zoster vaccine at a non-KP facility during the study period may not be complete. Of note, the information of vaccines received out of KPSC would be entered into the EMR, provided there is sufficient documentation. The vaccination information prior to their KPSC health plan enrollment might not be fully captured in EMR, and those subjects would be misclassified as being eligible for zoster vaccine and included in the study population, which could result in an underestimated vaccination rate. Although KPSC health plan members are unlikely to obtain vaccines at non-KP facilities because they would not receive reimbursement for vaccines received out of the KPSC health plans, the members who received vaccines at non-KP facilities during the study period might be misclassified as non-recipients which would also cause an underestimated vaccination rate. This potential bias in estimate of zoster vaccination rate could have occurred in both pre- and post- Tier-6 implementation periods. However we anticipated that the rate for misclassification is unlikely to vary substantially over time during the study period, thus this misclassification would not substantially bias the estimate for the trend of vaccination rates.

Another limitation is that as the Tier-6 was implemented in the entire KPSC region, we were unable to identify a concurrent comparison cohort of Medicare Part D members that were not affected by the Tier-6 implementation. To account for the impact of unknown secular trends in environmental factors (specific or non-specific for zoster vaccine) that might have affected the vaccination rate, such as shortage of zoster vaccine, DTC advertising, and general increasing public awareness of the zoster vaccine, or promotion of vaccines among elderly over time, we used two commercial health plan comparison cohorts. The zoster vaccine was covered for free to all commercial health plan members during the entire study period and the commercial health plan members also experienced similar changes in zoster vaccine supply, DTC advertising, and public awareness of zoster vaccine during the study period. However, vaccination rate change over time may also be affected by other factors including vaccination recommendations from physicians. This study did not measure physician recommendations. Since age could be associated with a higher risk of shingles and a stronger physician recommendation for this vaccine among older subjects, we used ≥65-year-old commercial health plan members as a comparison cohort to account for the potential age effect on vaccine uptake behavior.

## Conclusions

The impact of introduction of Tier-6 in the Medicare Part D benefit on zoster vaccination was not substantial in elderly Medicare Part D beneficiaries in an integrated health care organization which applied a lower than average copay ($20 to $40) prior to the Tier-6 implementation. Further research is needed to explore the association between the introduction of Medicare Part D Tier-6 and the zoster vaccine uptake among elderly in other health care settings where a higher cost-sharing/copay was applied prior to the implementation of Tier-6. In addition, the numerical relationship between zoster vaccination rate and the amount of patient copay can be explored in plans where the copay amount has a sufficient range of variation.

## Additional file

Additional file 1: Focus on the Patient. Patient-focused highlights providing the context, the outcomes and the impact of the study in the plainest language possible. (DOCX 13 kb)

## Abbreviations

ACIP: Advisory Committee on Immunization Practices
CI: Confidence intervals
CMS: Centers for Medicare & Medicaid Services
DID: Difference-in-difference
DTC: Direct-to-consumers
EMR: Electronic medical record
KPSC: Kaiser Permanente Southern California
NHIS: National Health Interview Survey
SES: Socioeconomic status; US, United States
