# Supplementary material for: Impact of the change of copay policy in Medicare Part D on zoster vaccine uptake among Medicare beneficiaries in a managed care organization
Source: BMC Health Serv Res. 2017 Jul 21;17:503. doi: 10.1186/s12913-017-2441-7 (PMC5521141; doi:10.1186/s12913-017-2441-7)
Supplement: Additional file 1: — Focus on the Patient. Patient-focused highlights providing the context, the outcomes and the impact of the study in the plainest language possible. (DOCX 13 kb) [file 12913_2017_2441_MOESM1_ESM.docx]

**FOCUS ON THE PATIENT**

- Zoster vaccine coverage remained low in elderly population after the routine recommendation.
- In 2011, Centers for Medicare & Medicaid Services (CMS) introduced a new tier (Tier-6) in Medicare Part D to cover the costs for all injectable Part D vaccines (including the zoster vaccine).
- To date, the impact of the implementation of Tier-6 on zoster vaccination uptake in persons with Part D coverage (those aged ≥65 years) has not been evaluated.
- The impact of Tier-6 on zoster vaccination was not substantial when a low copay ($20 to $40) was applied prior to the Tier-6 implementation.
- Further research is necessary to explore the numerical relationship between vaccination and amount of copay.
